# Toward a Molecular Classification of Colorectal Cancer: The Role of Telomere Length

**DOI:** 10.3389/fonc.2014.00158

**Published:** 2014-06-18

**Authors:** Esha Baichoo, Lisa A. Boardman

**Affiliations:** ^1^Division of Gastroenterology and Hepatology, Department of Medicine, Mayo Clinic, Rochester, MN, USA

**Keywords:** telomere attrition, telomerase, hTERT, colorectal cancer, cancer risk

## Abstract

Telomere biology is central to the maintenance of genomic stability and telomeric dysfunction is thought to be an early stage in carcinogenesis. Reports of telomere lengths and their ascribed colorectal cancer (CRC) risks have been discordant, with both very short and very long telomeres implicated. Nevertheless, telomeres appear to play a very central role in cancer initiation. Telomere length changes also appear to impact disease burden, progression, and overall survival. This review covers contemporary views on telomere biology and CRC risk, with a brief overview of analytical methods employed in telomere measurement. We conclude with arguments in favor of including telomere assessment in the molecular profiling of CRCs.

## Introduction

Telomeres are repeat TTAGGG sequences at the end of linear chromosomes, which guard against loss of genetic material during cellular replication. Due to an inherent end replication problem, chromosomes are exposed to a potential loss of genetic material, with telomeres acting as a buffer against loss of chromatin. Repeated cell cycles eventually lead to a critically shortened telomere length, signaling cellular senescence, and triggering apoptosis. This arrest in proliferation is thought to protect against malignant transformation and a failure to do so results in catastrophic genomic instability and carcinogenesis. Telomeres are thus important in managing genomic stability. This central role in genome maintenance makes telomeres key players in carcinogenesis and an attractive candidate for tumor profiling at the molecular level.

## Telomeres and Colorectal Cancer

**Figure 1:**
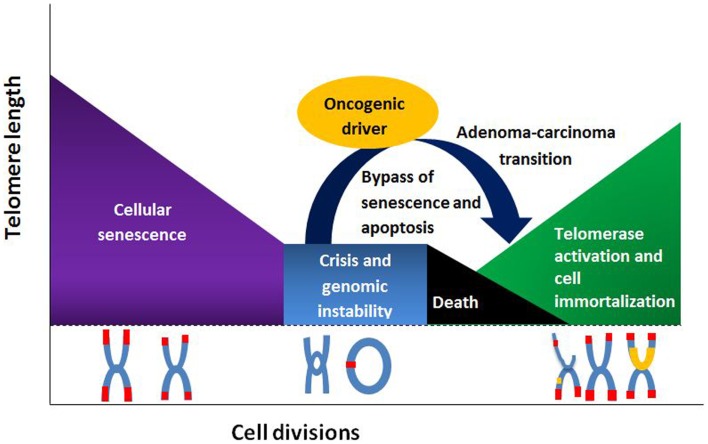
**Telomere length and its relationship to cell division, senescence, and senescence by-pass**.

Telomere length changes have been linked to numerous cancers, including colorectal cancer (CRC). Results from studies analyzing telomere lengths in CRC have been discordant, presenting evidence that both ends of the spectrum (shorter and longer lengths) have a possible role in CRC occurrence (1–3). Moreover, reports of null association have been described (1, 4, 5). Nevertheless, studies linking telomere attrition, or shortening, to an increase in CRC risk have classically dominated literature. Telomeric dysfunction is thought to represent an early step in many epithelial cancers (6). As telomeres reach their critical length, senescent signals are sent, and cells undergo cellular arrest and apoptosis. By-passing this senescent signal and cellular arrest results in continuous replication, with progressive telomere shortening. Eventually telomeres become so short that end-to-end fusions with structural and numerical chromosomal changes, anaphase bridging, and subsequent chromosomal instability ensue (7). This so-called telomere catastrophe halts further cellular divisions (8). However, in the presence of loss of tumor suppressor function, such as an APC mutation or p53 inactivation, pre-malignant cells are able to by-pass this event through telomere maintenance mechanisms. Telomerase, a ribonucleoprotein reverse transcriptase, stabilizes the telomere lengths, protecting the altered chromosomes, and immortalizing pre-malignant cells, thus enabling cancer progression (9, 10). This telomerase upregulation occurs at the critical point in the adenoma-carcinoma transition, allowing evasion of telomeric catastrophe, and supporting malignant progression (see Figure 1) (3, 11). Less-commonly, telomere length may be preserved through a recombination-dependent mechanism (12).

The tumor micro-environment may also contribute to carcinogenesis. Shortened telomeres in stromal cells may participate in epithelial changes leading to cancer via autocrine or paracrine mechanisms. Thus as stromal cells undergo senescence, they exhibit a secretory phenotype that may trigger neighboring cells with shortened telomeres to by-pass the senescence signal and termination, setting the stage for chromosomal instability and malignant transformation (13). This theory, known as the senescence-associated secretory pathway, could explain the finding that shortened telomere lengths in some polyps and CRC mirrored the shortened telomeres in the surrounding tissue, suggesting that a shortened telomere length may predate malignant transformation and is not a consequence of cancer progression (14). Chronic inflammation processes in surrounding epithelial cells, as in chronic ulcerative colitis, has also been linked to an increased telomere attrition rate and malignant progression, giving credence to the theory that telomere shortening in the micro-environment may act as a nidus for malignant transformation (15).

Epidemiological studies evaluating the relationship between telomere lengths and CRC risks have produced conflicting results. Although telomere attrition is classically thought of as a risk for CRC, reports of longer telomere lengths, and a predisposition to CRC have emerged (2, 16–18). To complicate matters, some studies report a duality of results, with both shorter and longer telomeres associated with increased CRC risk (1, 18). Interestingly, the findings of longer telomeres and their association to CRC seem prevalent in prospective studies, while retrospective studies report, for the most part, shorter telomere lengths. Pooley et al. report an association of shortened telomere lengths and CRC risk in retrospectively collected samples, but fail to replicate their results in the prospective arm of their study (1). Given the conflicting results with respect to study timeline, the argument of reverse causality has been made. Simply put, the changes in telomere lengths, especially in the case of shorter telomeres, may represent disease progression and/or therapeutic interference. Shorter telomere lengths could therefore be a marker of disease progression rather than one of causality. Moreover, the dual findings that both extremely short and long telomeres may be associated with CRC, sometimes within the same sample set, point to the possibility of a “healthy range” of telomere lengths within which cancer risk need not be increased.

## Assessment of Telomere Length

A multitude of analytical techniques are available for telomere length measurement, including Southern Blot, quantitative PCR (qPCR), flow cytometry with fluorescence *in situ* hybridization (flow-FISH), quantitative FISH (Q-FISH), and single or universal single telomere length analysis (STELA) (19). Southern Blot and qPCR are the two most commonly used techniques in epidemiological studies assessing peripheral blood leukocyte (PBL) telomere length. Southern Blot analysis is the gold standard for telomere length assessment, providing results, in terminal restriction fragment (TRF) units, that are deemed reproducible and that allow inter-study comparisons. However, Southern Blot analysis requires a large amount of high-quality DNA and may exaggerate telomere length by including subtelomeric DNA (20). Furthermore, the type of restriction endonuclease used may impact measurement results (20). Cancer risk association studies typically use qPCR to determine telomere lengths. qPCR uses primers to the telomeric repeats to amplify telomeres (21). The telomere to single copy gene ratio of the sample is then compared to the ratio of a reference DNA sample, yielding a relative telomere length (21). qPCR does not require a large amount of DNA but inter-laboratory differences in the reference DNA limit comparisons between studies. The estimated variability between assays is >6% for qPCR and >2% for Southern Blot (22). Telomerase activity may also be measured by way of the Telomere Repeat Amplification Protocol (TRAP), which utilizes PCR to amplify telomerase-extended primer products (23). Alternatively, telomerase activity can be measured via a direct primer extension activity assay, which has proven effective in cell assays but has as yet to be applied to tissues. This direct extension assay avoids the limitations of the TRAP assay, which can be less accurate due to non-linear amplification and due to false negative results in the presence of inhibitors of Taq DNA polymerase (24). hTERT mRNA expression using real-time PCR has also been used to characterize telomerase activity. hTERT mRNA expression is thought to provide a good correlation with telomerase activity in certain cancers, including CRC and has been suggested to have a negative prognostication value in CRC (25, 26). Given the wide range of analytical tools available, it is not unreasonable to assume that some of the discrepancies in published results may be due to measurement error and inter-laboratory variation. In a large study, Cunningham et al. find that DNA extraction methods greatly influenced telomere length readings (27). A standardized extraction and measurement method is therefore imperative to allow comparison and validation of published data.

## Telomeres and Molecular Subclassification of CRC

The lack of consensus on telomere length changes and the conferred risk for CRC could point toward the existence of distinct molecular subclasses of CRC. Telomere dysfunction may represent an alternative pathway in CRC carcinogenesis, a shift from the two classic genomic instabilities observed: chromosomal and microsatellite instability (MSI). In a study assessing telomere attrition in microsatellite stable (MSS) and chromosomally stable (CIN−) rectal cancers, Boardman et al. revealed evidence of molecular heterogeneity within MSS cancers, in regards to their CIN status and telomere maintenance mechanism (16). Of interest was the discovery of a subset of MSS and CIN-rectal cancers with the unique molecular profile of increased alternative lengthening pathway (ALT+) and longer telomere lengths, in contrast to the shortened telomeres and increased telomerase expression in the chromosomally unstable (CIN+) subgroup (16). Although ALT expression has been described in various cancers, its association to CRC is not clearly defined (28, 29). ALT offers a distinct telomere maintenance mechanism from telomerase, involving superimposed lengthening and shortenings in a recombination-dependent fashion (30). The results are long, heterogeneous telomeres, and the pathognomonic ALT associated promyelocytic leukemia bodies (APBs) (28). From this perspective, the distinct molecular profiles observed could point to the existence of a different molecular subclass of CRC. In a separate study, the authors also find an age at onset-dependent difference in PBL telomere length changes and CRC risk (31). Longer PBL telomeres appear to be a predictor of CRC in their young-onset CRC subgroup ( ≤50 years old) while extremely short PBL telomeres are associated with CRC in older individuals. While shorter telomere lengths in the older subgroup ( ≥50-year-olds) may partly be explained by the natural aging process and resultant chromosomal instability, the association of longer telomeres and CRC risk in younger patients may suggest genetic alterations in telomere maintenance mechanisms. While reverse causality, or the effect of disease burden and therapy, may certainly be an explanation for the longer telomere lengths observed, the result may also indicate a diverging telomere-centered mechanism, and potentially, a distinct molecular subclass of CRC with early penetrance.

Studies looking into the relationship between MSI and telomere lengths have been sparse. Telomeres may be considered a form of super microsatellite given their tandem repeat nature and it may be reasonable to assume that defects in MMR genes impact telomere length. In an experiment studying the effect of down-regulating MSH2 in fibroblast cell lines, Mendez-Bermudez et al. report a statistically significant increase in telomere attrition rate compared to control cell lines (32). However, we do not fully understand how these findings might translate into the prognosis and treatment of patients with dMMR CRC.

The impetus for adding telomere lengths to the molecular profiling of CRC lies in the possibility of tailored therapy. Differences in molecular profiles often translate into distinct clinical progression, as in the case for CIN− and CIN+ CRC. Genetic CRC may also benefit from a telomeric subclassification, as telomere length changes have been suggested as risk modifiers in mutation carriers and therefore may serve as a marker of prediction (33). The association between telomere length and disease progression has been described, both in early and more advanced cases (34, 35). Riegert-Johnson et al. report shorter PBL telomere in individuals with advanced polyps compared to age- and gender-matched polyp-free individuals, suggesting that PBL telomere be used as a biomarker for advanced adenomatous polyps (34). This biomarker for stratifying patients according to their progression risks could serve as a pre-screening step, identifying patients needing more frequent colonoscopic surveillance. Telomerase activity has also been suggested as a marker for progression, with numerous reports on the association between increased telomerase expression and malignant transformation (3, 11). Telomerase activity appears to correlate to disease stage, with Duke A and B stages expressing lower telomerase activity (36). Furthermore, telomerase activity reflected disease burden, risk of recurrence, and overall survival (11, 26, 37). Thus, quantifying telomere length and telomerase activity may serve as a useful prognostication tool.

There has been intense interest in anti-telomerase drugs and their potential as a targeted chemotherapeutic drug (38, 39). Targeted therapy could reduce treatment-resistance and side-effects. The combination of a telomere-centered subclassification and targeted treatment could translate into individualized medical care and better patient outcomes. Determining telomeric molecular profiles and their ascribed cancer risk also opens the possibility for chemoprevention.

## Concluding Remarks

Very like the chicken or egg causality dilemma, the timeline of events involving telomeres, genomic instability, and CRC remain unclear. While distinct molecular mechanisms are conceivable, synergistic effects between different components and the interactions of alternative pathways cannot be ignored. Differences in analytic measures have been implicated in the varying results seen suggesting the need for a standardized measurement technique. The difference in results could also reflect a reverse-causation effect. Larger prospective studies are imperative to validate previously published data. Moreover, given the duality in findings, a healthy range of telomere lengths may need to be established. Nevertheless, a subclassification of CRC to include telomere status could carry significant value in predicting disease severity, progression and overall prognosis, and in directing treatment. A telomere subclassification represents an important step forward in individualized medicine and is, therefore, an important avenue to explore.

## Conflict of Interest Statement

Lisa A. Boardman is supported by the National Cancer Institute (RO-1 CA132718 and RO-1 CA 170357) and the National Institute of Diabetes and Digestive and Kidney Diseases (P30 DK084567 Mayo Clinic Center for Cell Signaling in Gastroenterology). The authors have no other relevant affiliations or financial involvement with any organization or entity with a financial interest in or financial conflict with the subject matter or materials discussed in the manuscript apart from those disclosed.
